# Monoclonal Antibodies Targeting IL-5 or IL-5Rα in Eosinophilic Chronic Obstructive Pulmonary Disease: A Systematic Review and Meta-Analysis

**DOI:** 10.3389/fphar.2021.754268

**Published:** 2021-11-02

**Authors:** Chuchu Zhang, Yalei Wang, Meng Zhang, Xiaojie Su, Ting Lei, Haichuan Yu, Jian Liu

**Affiliations:** ^1^ Department of Intensive Care Unit, Lanzhou University First Affiliated Hospital, Lanzhou, China; ^2^ The First Clinical Medical College of Lanzhou University, Lanzhou University, Lanzhou, China

**Keywords:** eosinophils, monoclonal antibodies, anti-IL-5, COPD, meta-analysis

## Abstract

**Background:** Although the predominant airway inflammation in chronic obstructive pulmonary disease (COPD) is neutrophilic, approximately 20–40% of COPD patients present with eosinophilic airway inflammation. Compared with non-eosinophilic COPD patients, eosinophilic COPD patients are characterized by a greater number of total exacerbations and higher hospitalization rates. Furthermore, anti-interleukin-5 (IL-5) therapy, consisting of monoclonal antibodies (mAbs) targeting IL-5 or IL-5 receptor α (IL-5Rα), has been proven to be effective in severe eosinophilic asthma. This meta-analysis aimed to determine the efficacy and safety of anti-IL-5 therapy in eosinophilic COPD.

**Methods:** We searched the PubMed, Web of Science, Embase, and Cochrane Library databases from inception to August 2020 (updated in June 2021) to identify studies comparing anti-IL-5 therapy (including mepolizumab, benralizumab, and reslizumab) with placebo in eosinophilic COPD patients.

**Results:** Anti-IL-5 therapy was associated with a decrease in acute exacerbation rate (RR 0.89; 95% CI 0.84 to 0.95, *I* ^*2*^ = 0%) and the severe adverse events (RR 0.90; 95% CI 0.84 to 0.97, *I* ^*2*^ = 0%). However, no significant improvement was observed in pre-bronchodilator forced expiratory volume in 1 s (FEV_1_) (WMD 0.01; 95% CI −0.01 to 0.03, *I* ^*2*^ = 25.9%), SGRQ score (WMD −1.17; 95% CI −2.05 to −0.29, *I* ^*2*^ = 0%), and hospital admission rate (RR 0.91; 95% CI 0.78 to 1.07, *I* ^*2*^ = 20.8%).

**Conclusion:** Anti-IL-5 therapy significantly reduced the annual acute exacerbation rate and severe adverse events in eosinophilic COPD patients. However, it did not improve lung function, quality of life, and hospitalization rate.

## Introduction

Chronic obstructive pulmonary disease (COPD) is characterized by progressive and irreversible airflow limitation that is triggered by the response of the airways and the lungs to noxious particles or fumes (Dave and Arjun, 2021). It is a leading cause of chronic morbidity and mortality worldwide (Dave and Arjun, 2021). COPD is a heterogeneous disease with different underlying pathobiological mechanisms (endotypes) and includes pulmonary and extra-pulmonary symptoms (phenotypes) (Han et al., 2010; Lange et al., 2016; Balkissoon, 2018; Dave and Arjun, 2021). Furthermore, as of May 2015, 99.9 million individuals suffering from COPD have been identified in China (Wang et al., 2018a). With continued exposure to COPD risk factors and an aging population, the prevalence of COPD is expected to increase over the next 40 years, and by 2060, more than 5.4 million may die from COPD and related conditions annually (Mathers and Loncar, 2006; Dave and Arjun, 2021).

Moreover, the exacerbation of COPD is associated with increased healthcare costs (Hilleman et al., 2000; Toy et al., 2010), progressive loss of lung function, subsequent cardiovascular events, and decline in quality of life (Dransfield et al., 2017; Kunisaki et al., 2018). Currently, Global Initiative for Chronic Obstructive Lung Disease (GOLD) guidelines have recommended triple inhaled therapy (inhaled glucocorticoids, long-acting β2-agonists, and long-acting muscarinic-receptor antagonists) as maintenance treatment for patients with frequent exacerbations, which was proven to decrease acute exacerbation rates in COPD patients (Calzetta et al., 2019; Dave and Arjun, 2021). Despite this, approximately 30–40% of patients continue to have moderate or severe exacerbations even after receiving triple inhaled therapy (Vestbo et al., 2017). Thus, it is essential to explore new treatment options for COPD patients with acute exacerbation.

Compared with non-eosinophilic COPD patients, eosinophilic COPD patients are associated with a higher number of total exacerbations and higher hospitalization rates (Couillard et al., 2017). Saha et al. have reported that 20–40% of COPD patients presented with airway eosinophilic inflammation (peripheral blood eosinophil count of 3% or more or >150 cells per cubic millimeter) (Dasgupta et al., 2013; Singh et al., 2014), although the predominant airway inflammation in COPD is neutrophilic (Hogg et al., 2004; Dasgupta et al., 2013). Interleukin-5 (IL-5) regulates the differentiation, proliferation, survival, and activation of eosinophils *via* the IL-5 receptor (Takatsu et al., 1994). Anti-IL-5 therapy includes monoclonal antibodies (mAbs) targeting IL-5 or IL-5R α (including mepolizumab, benralizumab, and reslizumab), which have been proven to be effective in severe eosinophilic asthma (Farne et al., 2017). Given the similarity between asthma and COPD in terms of eosinophilic airway inflammation, several randomized controlled trials (RCTs) have studied the efficacy and safety of anti-IL-5 treatment in eosinophilic COPD patients (Brightling et al., 2014; Dasgupta et al., 2017; Sciurba et al., 2018; Criner et al., 2019).

However, contrasting results on the efficacy of anti-IL-5 therapy to reduce annual exacerbation rates of eosinophilic COPD have been reported. Pavord et al. have found that treatment with mepolizumab was associated with a lower incidence of moderate and severe exacerbations than placebo (Sciurba et al., 2018). In contrast, Brightling et al. and Criner et al. have noted that benralizumab did not reduce the annual exacerbation rates compared with the placebo (Brightling et al., 2014; Criner et al., 2019). Takudzwa et al. have conducted a meta-analysis and demonstrated that mepolizumab decreased the exacerbation rate by 23% in COPD patients with eosinophil counts of 300 cells/μL or greater than controls. (Mkorombindo and Dransfield, 2019). The efficacy of anti-IL-5 therapy in eosinophilic COPD is therefore not consistent.

Although the meta-analysis on anti-IL-5 in COPD patients already existed (Donovan et al., 2020; Lan et al., 2020), study participants were not limited to eosinophilic COPD patients. To provide more accurate and stronger evidence for the efficacy of anti-IL-5 therapy in eosinophilic COPD patients, the current study differs in two ways from the previous meta-analysis (Dave and Arjun, 2021): we only included eosinophilic COPD patients (peripheral blood eosinophil count of 3% or more or >150 cells per cubic millimeter) (Balkissoon, 2018); we compared anti-IL-5 therapy in eosinophilic COPD and in asthma, which enabled a more robust assessment of the effect of anti-IL-5 therapy in eosinophilic COPD patients.

## Methods

This meta-analysis followed the guidelines of the Cochrane Handbook for Systematic Reviews of Interventions. Furthermore, we conducted this meta-analysis according to the Preferred Reporting Items for Systematic Reviews and Meta-analysis (PRISMA) guidelines (Moher et al., 2009). The protocol for this meta-analysis is available in PROSPERO (CRD42020156189) (Wang et al., 2018b; Ge et al., 2018).

### Literature Search

We searched the PubMed, Web of Science, Embase, and Cochrane Library databases from inception to August 2020 (updated in June 2021) to identify studies comparing anti-IL-5 therapy (including mepolizumab, benralizumab, and reslizumab) with placebo in COPD patients. There was no language or population restriction. In addition, we searched the ClinicalTrials.gov database to identify completed studies. We used the following keywords to perform the search: monoclonal antibody (mepolizumab, benralizumab, and reslizumab) and chronic obstructive pulmonary disease. We have displayed the detailed search strategy in Supplementary Material.

### Inclusion and Exclusion Criteria

Inclusion criteria were as follows:

1. RCTs included parallel group studies, had a controlled design, and compared anti-IL-5 therapies with placebo.
2. Studies were conducted in adult patients with eosinophilic COPD, defined as peripheral blood eosinophil count of 3% or more or >150 cells per cubic millimeter.
3. Intervention was restricted to anti-IL-5 therapy or placebo.
4. Study outcomes were required to be at least one of the following: annual exacerbations, hospital admission for acute exacerbation, improvement of pre-bronchodilator forced expiratory volume in 1 s (FEV_1_), quality of life as assessed using the St. George’s Respiratory Questionnaire (SGRQ) total score, and severe adverse events.

Exclusion criteria were as follows:

1. Studies including participants who suffered from clinically significant lung disease or asthma.
2. Conference abstracts, letters, comments, reviews, and meta-analyses.
3. Studies of animals or cells.

### Study Selection and Data Extraction

Author CZ screened all titles and assessed full-text eligibility and then excluded studies that did not meet the inclusion and exclusion criteria. Author YW reassessed the selection results; all discrepancies were resolved by discussing them with a third author MZ. Two authors (XS and TL) independently extracted the following data from all included studies: lead author or study title, year of publication, location and duration, demographic characteristics of participants, drug and dose of anti-IL-5 therapy, annual exacerbations, hospital admission for acute exacerbation, change of pre-bronchodilator FEV_1_ from baseline, SGRQ score, and severe adverse events. Disagreements were settled by cross-checking original papers and consensus was achieved. Author HY validated and sorted specific data in a tabular format. The primary outcome was annual exacerbations, as acute exacerbation is a major cause of hospitalization and poor prognosis in COPD. The secondary outcomes were hospital admission for acute exacerbation, pre-bronchodilator FEV_1_, SGRQ score, and severe adverse events.

### Assessment of Risk of Bias in Included Studies

Two authors (CZ and XS) independently evaluated the quality of the methodology of the eligible RCTs. They applied the Cochrane Collaboration tool following the Cochrane Handbook for Systematic Reviews of Interventions (Stovold et al., 2014). There were six perspectives used to assess the quality, including random sequence generation (selection bias), allocation concealment (selection bias), blinding (performance bias and detection bias), incomplete outcome data (attrition bias), selective outcome reporting (attrition bias), and other potential sources of bias. The criteria to grade the included studies were as follows: 1) low-quality trial: either randomization or allocation concealment was assessed to indicate a high risk of bias, regardless of other items; 2) high-quality trial: both randomization and allocation concealment were graded as low risk of bias, and all other items were assessed as low or unclear risk of bias; 3) moderate-quality trial: they did not meet the criteria for high or low risk. Any discrepancy was resolved by consulting an evidence-based medicine professor.

### Statistical Analysis

Stata/SE 15.0 was used to perform data analysis. We pooled the rate ratio (RR) and 95% confidence interval (CI) to analyze the overall annual exacerbation rates. Dichotomous data, including hospital admission rate, severe adverse events, and all-cause mortality, were analyzed by calculating risk ratios (RR) and the corresponding 95% CI. Continuous data (pre-bronchodilator FEV_1_ and SGRQ scores) were analyzed by calculating the weighted mean difference (WMD) or standardized mean difference (SMD) and 95% CI. We used *P* and I^2^ statistics to measure heterogeneity among trials in each analysis. Fixed-effects models were used without important heterogeneity (I^2^ ≤ 50%). Otherwise, random effects models were used. A funnel plot was generated for examining publication bias when there were >10 included trials (Lau et al., 2006; Stovold et al., 2014). A *p*-value <0.05 was considered statistically significant.

## Results

### Eligible Studies and Risk of Bias

**FIGURE 1:**
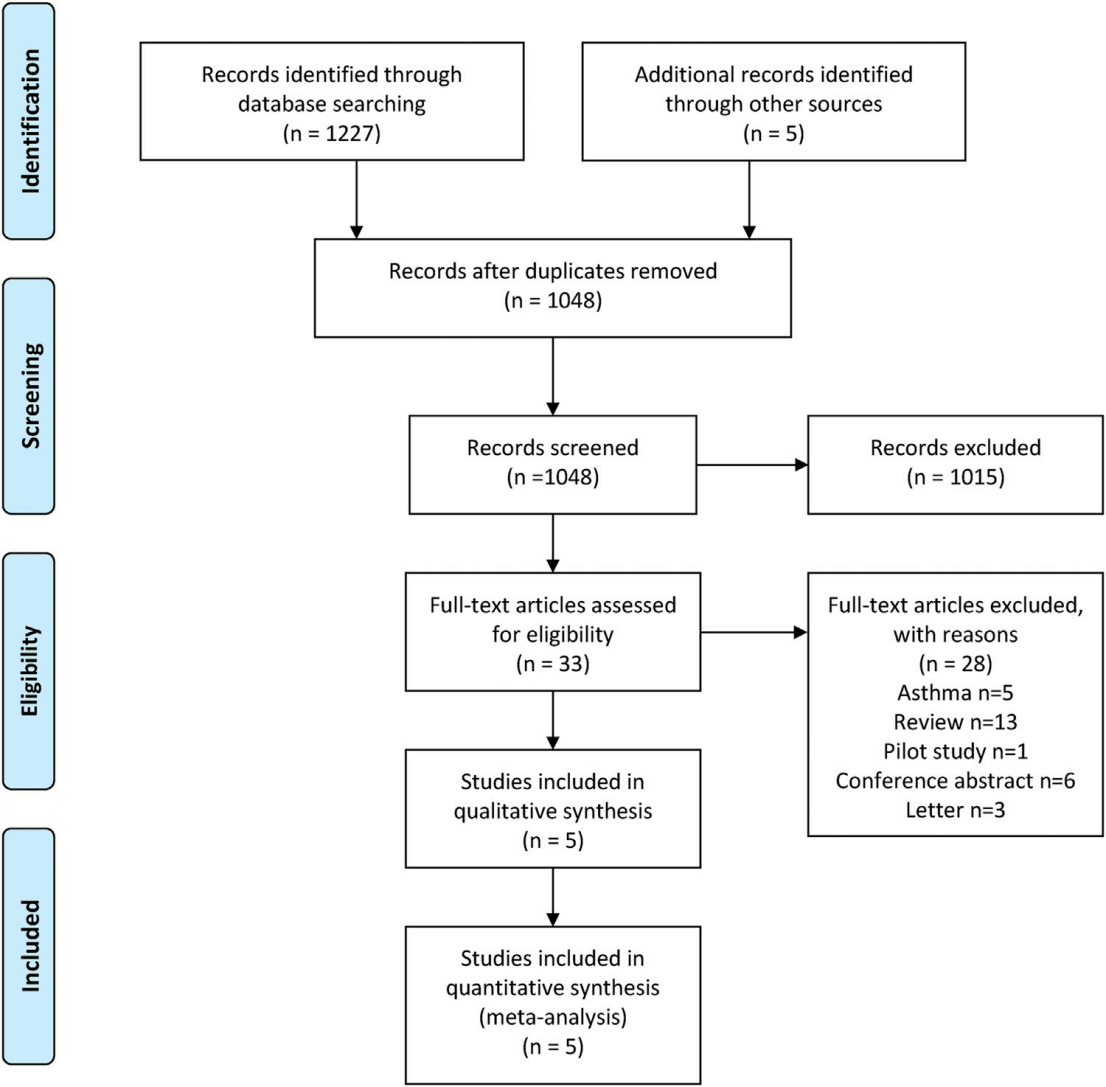
Study selection process: PRISMA flow diagram identifying studies included in the meta-analysis. PRISMA, Preferred Reporting Items for Systematic Reviews and Meta-Analyses.

We obtained 1,227 articles from the four databases and five studies from the ClinicalTrials.gov database. After removing the duplicates, 1,048 articles remained. We excluded 1,015 articles after scanning the titles and abstracts. Finally, three articles, including five studies, were included in this meta-analysis after reading the full text (Criner et al., 2019; Sciurba et al., 2018; Brightling et al., 2014). The detailed selection process is shown in Figure 1, which was prepared based on the PRISMA guidelines (Moher et al., 1996). Three studies were rated as high quality based on the grade criteria, the six items of the Cochrane tool shown in Supplementary Figures S1, S2.

### Description of Eligible Studies

All included studies were randomized, double-blinded, multicentered RCT, aiming to compare the clinical efficacy and safety of anti-IL-5 therapy with those of the placebo in adult patients with eosinophilic COPD. In the included five studies, the intervention was performed with benralizumab (10, 30, and 100 mg) targeting the IL-5 receptor α (20, 22) or mepolizumab (100 and 300 mg) targeting IL-5 (Brightling et al., 2014; Criner et al., 2019). Overall, there were 3902 COPD patients included in this meta-analysis. Current smoker status ranged from 25 to 42% among the study population and 58.0–70.7% of the patients were males. We have listed the detailed baseline characteristics in Table1.

**TABLE.1:** Characteristic of studies included in this meta-analysis.

Study	Year	N	Age	Male%	Smoker%	Baseline EOS	Intervention	Duration	Outcome
Brightling	2014	101	62.9 ± 8.2/64.6 ± 7.5	68.6/58.0	33/42	248.8 ± 193.4/229.2 ± 164.5	B 100 mg	56	①②③④⑤
GALATHEA	2019	1,120	65.6 ± 8.25	70.7	34.3	453.2 ± 280.25	a. B 100 mg	56	①②③④⑤
							b. B 30 mg		
TERRANOVA	2019	1,545	65.2 ± 8.33	66.3	28.6	504.5 ± 393.08	a. B 100 mg	56	①②③④⑤
							b. B 30 mg		
							c. B 10 mg		
METREX	2017	462	66 ± 9/65 ± 9	62/63	25/28	260 ± 0.438/290 ± 0.558	M 100 mg	52	①③⑤
METREO	2017	674	65 ± 9/66 ± 9	59/69	25/28	300 ± 0.520/310 ± 0.515	a. M 100 mg	52	①③⑤
			65 ± 9/66 ± 9	70/69	32/28	310 ± 0.540/310 ± 0.515	b. M 300 mg		

Outcome: ① annual rate of acute exacerbation; ② change from baseline of pre-bronchodilator FEV_1_; ③ change from baseline of SGRQ total score; ④ hospital admission rate for acute exacerbation; ⑤ severe adverse events. B: benralizumab; M: mepolizumab.

### Annual Rate of Acute Exacerbation

**FIGURE 2:**
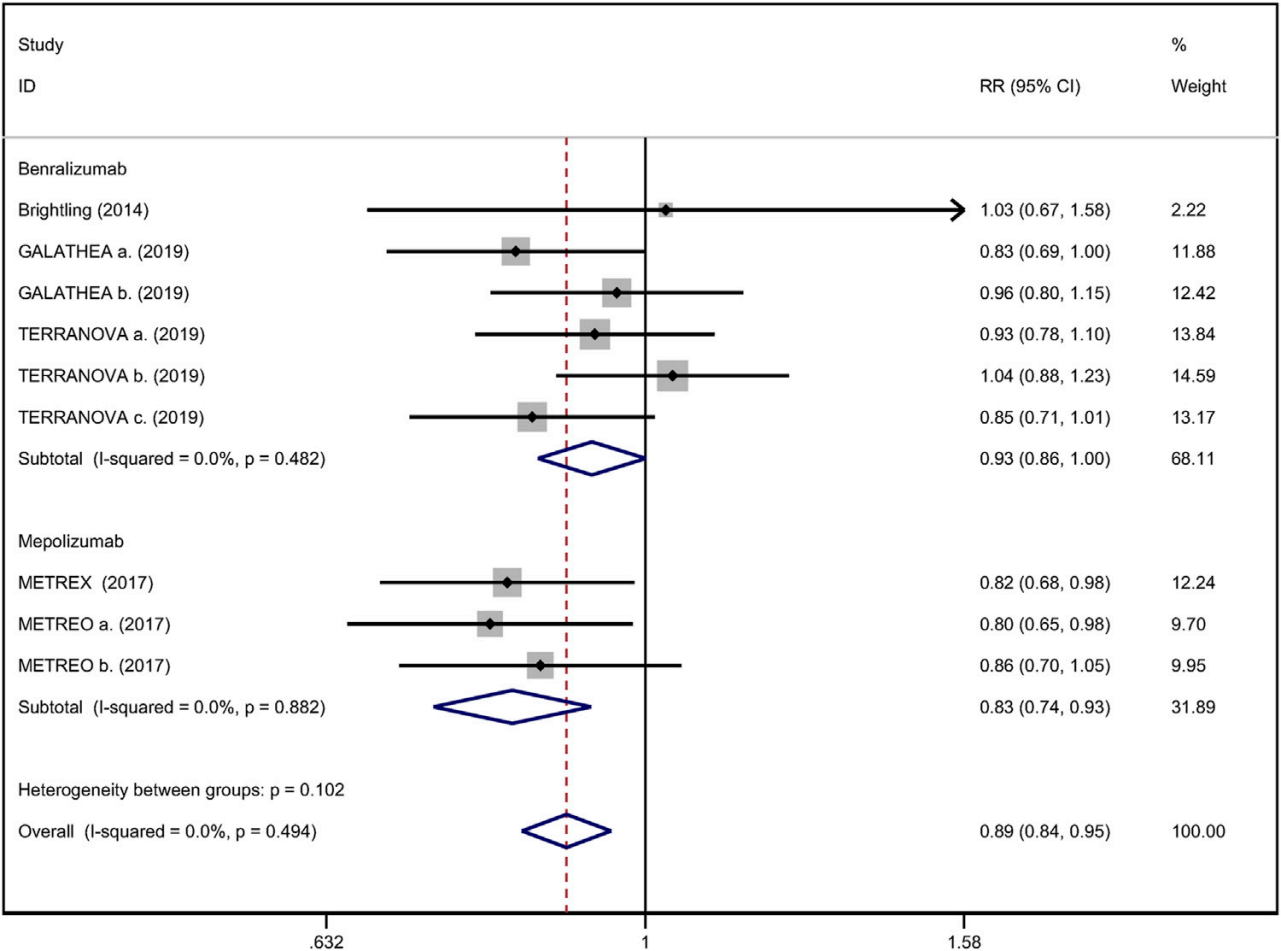
Forest plot of annual acute exacerbation rates in eosinophilic COPD patients with anti-IL-5 therapy vs. placebo.

All included studies reported the annual rate of exacerbations. There were five RCTs (Brightling et al., 2014; Sciurba et al., 2018; Criner et al., 2019) that compared anti-IL-5 therapy with placebo, showing that anti-IL-5 therapy was associated with a lower risk of acute exacerbation rate of eosinophilic COPD patients (RR 0.89; 95% CI 0.84 to 0.95, *I* ^*2*^ = 0%; Figure 2).

### Secondary Outcomes

**FIGURE 3:**
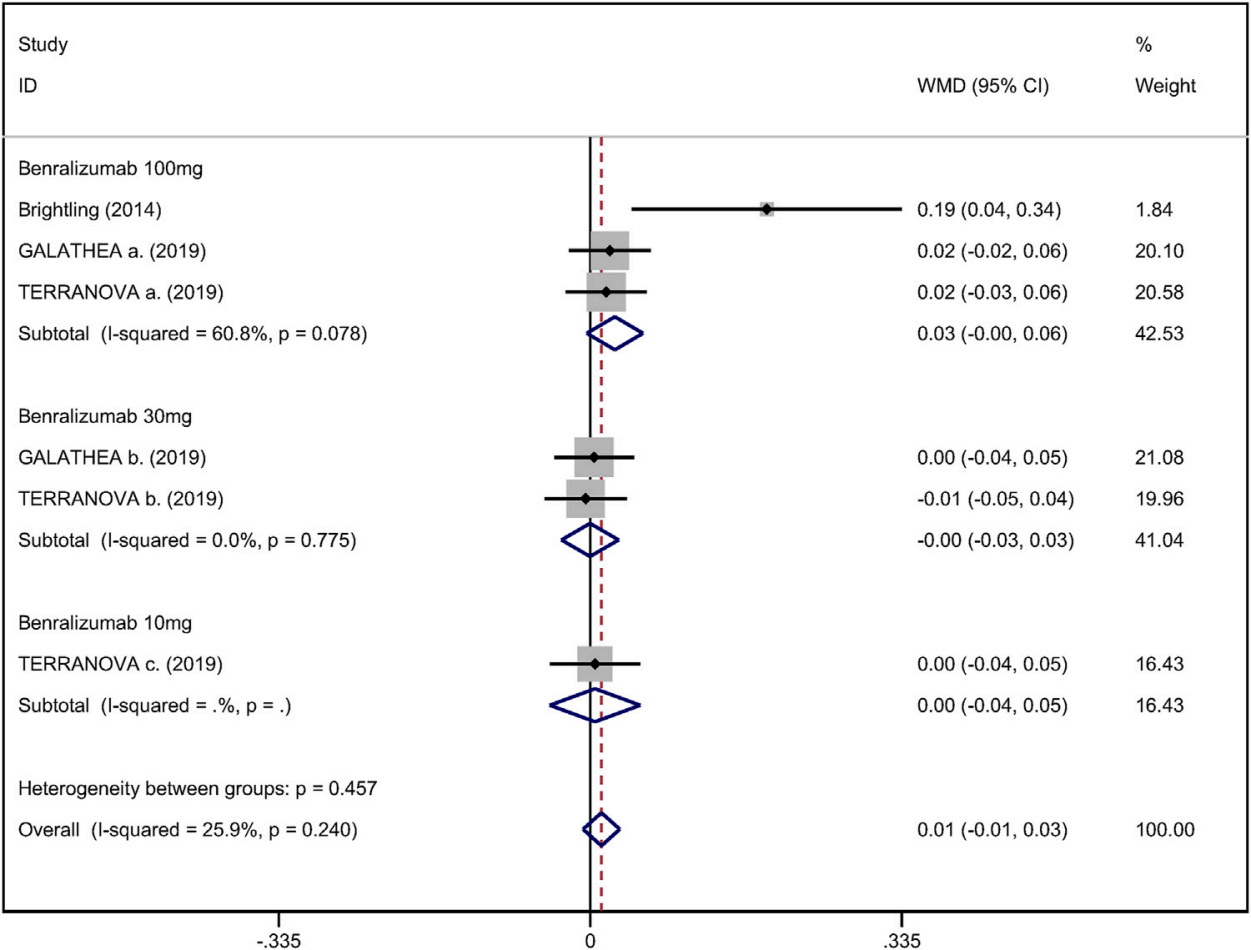
Forest plot of pre-bronchodilator FEV_1_ in eosinophilic COPD patients with anti-IL-5 therapy vs. placebo.

**FIGURE 4:**
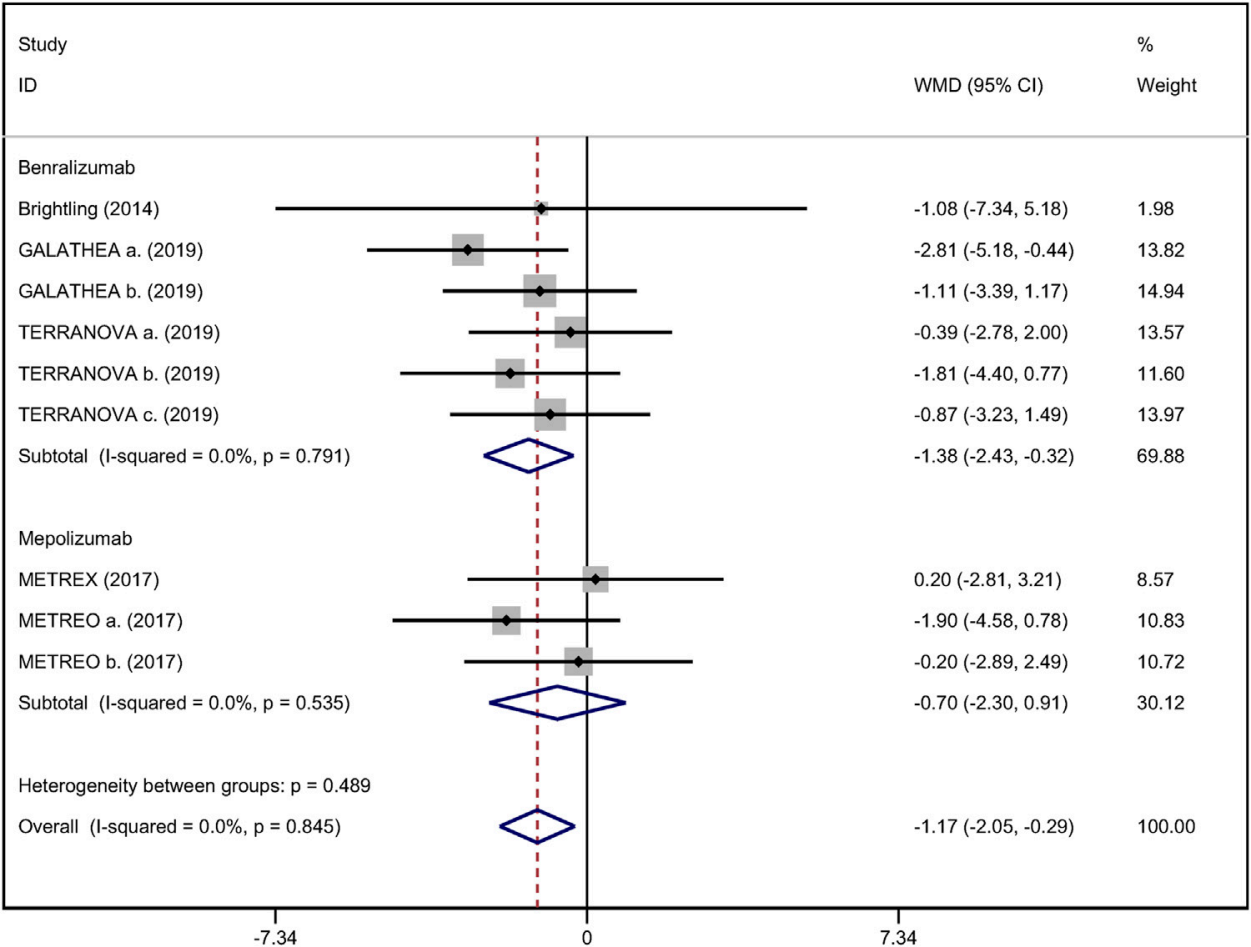
Forest plot of SGRQ score in eosinophilic COPD patients with anti-IL-5 therapy vs. placebo.

**FIGURE 5:**
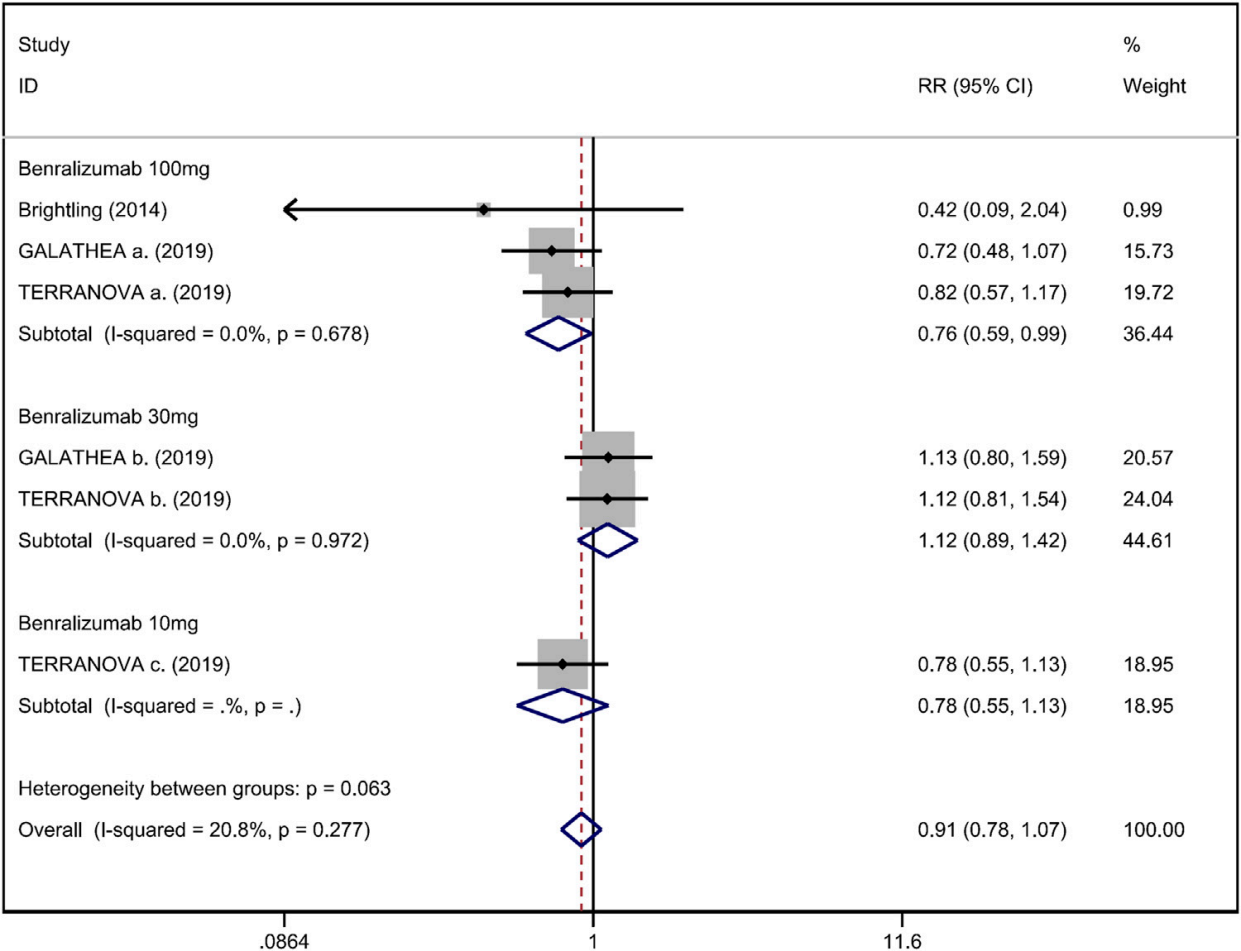
Forest plot of hospital admission rate for acute exacerbation in eosinophilic COPD patients with anti-IL-5 therapy vs. placebo.

**FIGURE 6:**
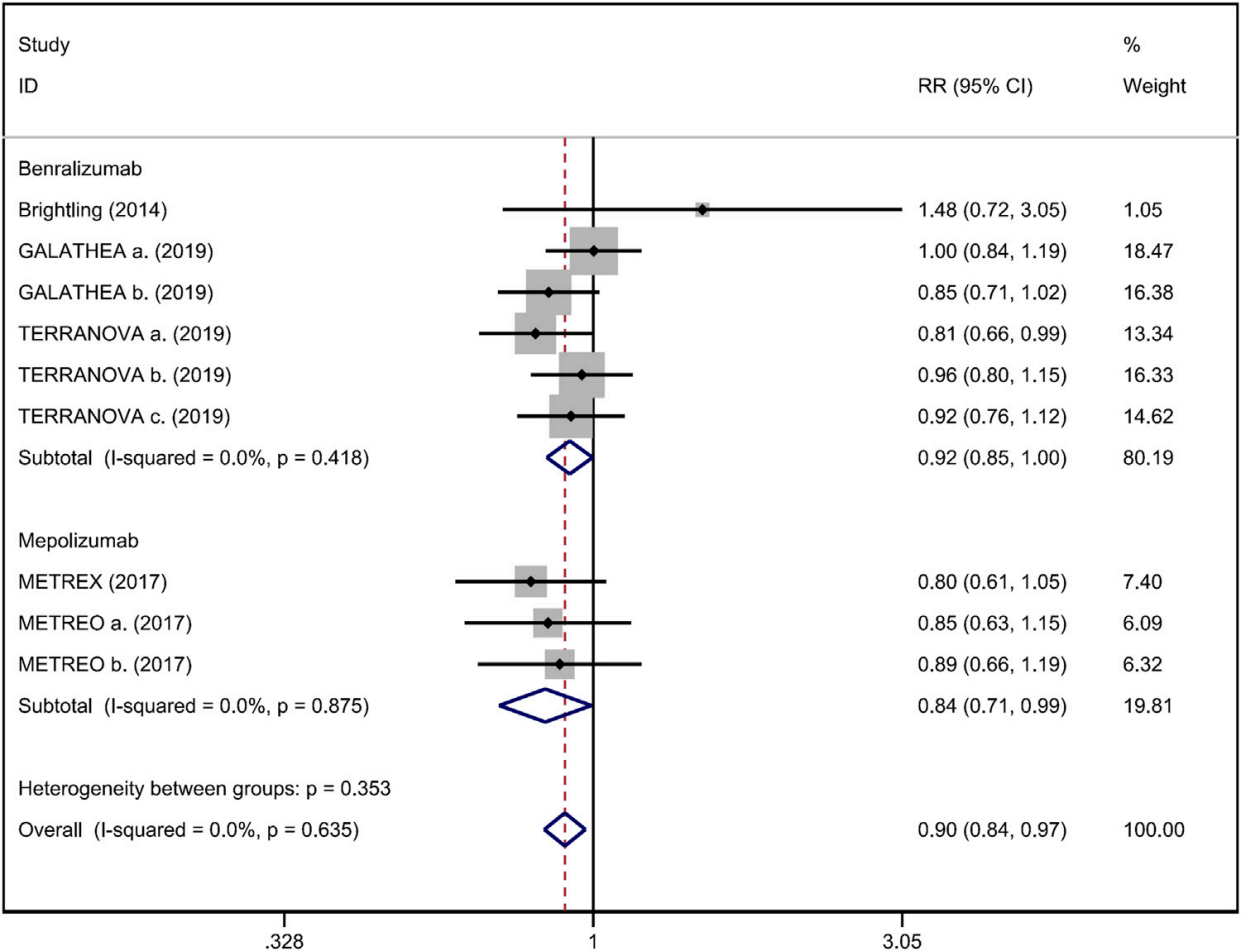
Forest plot of severe adverse event in eosinophilic COPD patients with anti-IL-5 therapy vs. placebo.

Mean change from baseline of pre-bronchodilator FEV_1_ was used to assess lung function. Three RCTs reported an improvement in FEV_1_. However, no significant difference between anti-IL-5 therapy and placebo with regard to pre-bronchodilator FEV_1_ was observed (WMD 0.01; 95% CI −0.01 to 0.03, *I* ^*2*^ = 25.9%; Figure 3) (Criner et al., 2019; Brightling et al., 2014). Improvement in quality of life was evaluated by the SGRQ total score, with a threshold of 4 units being considered clinically significant (Jones, 2005). Five RCTs reported changes in SGRQ total score. Anti-IL-5 was not associated with a significant improvement in the quality of life compared with placebo (WMD −1.17; 95% CI −2.05 to −0.29, *I* ^*2*^ = 0%; Figure 4) (Brightling et al., 2014; Sciurba et al., 2018; Criner et al., 2019). In addition, we assessed the hospital admission for acute exacerbation (Brightling et al., 2014; Criner et al., 2019). There was no significant difference in hospitalization rate between the anti-IL-5 therapy group and the placebo group (RR 0.91; 95% CI 0.78 to 1.07, *I* ^*2*^ = 20.8%; Figure 5). Regarding safety outcomes, the anti-IL-5 group demonstrated a significantly lower risk in the incidence of severe adverse events compared with the placebo group (RR 0.90; 95% CI 0.84 to 0.97, *I* ^*2*^ = 0%; Figure 6) (Brightling et al., 2014; Sciurba et al., 2018; Criner et al., 2019).

### Comparison With Anti-IL-5 Therapy in Asthma

To enrich our study, we compared the efficacy of anti-IL-5 therapy in eosinophilic COPD and asthma (Farne et al., 2017; He et al., 2018). The outcomes (including annual exacerbation rate, the pre-bronchodilator FEV_1_, the health-related quality of life, and the severe adverse events) of anti-IL-5 therapy on eosinophilic COPD or asthma are listed in Table 2. Anti-IL-5 therapy was significantly more effective in reducing the annual exacerbation rate in asthma patients than in eosinophilic COPD patients. Similarly, anti-IL-5 therapy showed a more remarkable improvement of pre-bronchodilator FEV_1_ in asthma patients than in eosinophilic COPD. Furthermore, mepolizumab led to a significant enhancement of health-related quality of life (by SGRQ score) in asthma but not in eosinophilic COPD. Finally, mepolizumab caused a more significant reduction of severe adverse events in asthma than in eosinophilic COPD.

**TABLE 2:** Comparison of anti-IL-5 between eosinophilic COPD and asthma.

Outcome	Eosinophilic COPD		Asthma	
	Benralizumab	Mepolizumab	Benralizumab	Mepolizumab
Annual exacerbation rate	0.93 (0.86, 1.00)	0.83 (0.74, 0.93)	0.62 (0.55, 0.70)	0.45 (0.36, 0.55)
Pre-bronchodilator FEV_1_	0.01 (−0.01, 0.03)	NA	0.10 (0.05, 0.14)	0.11 (0.06, 0.17)
Health-related quality of life				
SGRQ	−1.38 (−2.43, −0.32)	−0.70 (−2.30, 0.91)	NA	−7.40 (−9.50, −5.29)
ACQ	NA		−0.20 (−0.29, −0.11)	−0.42 (−0.56, −0.28)
AQLQ			0.23 (0.11, 0.35)	NA
Severe adverse events	0.92 (0.85, 1.00)	0.84 (0.71, 0.99)	0.81 (0.66, 1.01)	0.63 (0.41, 0.97)

ACQ, asthma control questionnaire; AQLQ, asthma quality of life questionnaire; SGRQ, St George’s respiratory questionnaire.

## Discussion

In this meta-analysis, we assessed the efficacy and safety of anti-IL-5 therapy in eosinophilic COPD patients. Several key findings were obtained: anti-IL-5 therapy significantly reduced the annual exacerbation rates without increasing the occurrence of severe adverse events (Brightling et al., 2014; Sciurba et al., 2018; Criner et al., 2019). However, the anti-IL-5 group did not show a significant improvement with regard to lung function, quality of life, and hospitalization (Brightling et al., 2014; Sciurba et al., 2018; Criner et al., 2019).

This meta-analysis demonstrated that anti-IL-5 therapy decreased the acute exacerbation rate in eosinophilic COPD patients. This result has physiological plausibility. IL-5 is a well-researched cytokine in eosinophilic inflammation, which is particularly vital for the differentiation, proliferation, and activation of eosinophils. It is released by the following 3 cells: CD4^+^ Th2 lymphocytes, eosinophils, and innate lymphoid cells. Both eosinophils and basophils express the IL-5R (Bagnasco et al., 2017; Yousuf et al., 2019). Mepolizumab reduces eosinophil counts in the blood and tissues by avidly binding to IL-5, preventing IL-5 from binding to eosinophil surface receptors (Hart et al., 2001; Varricchi et al., 2016). Benralizumab enhances antibody-dependent cell-mediated cytotoxic effects by binding to IL-5Rα, in turn reducing sputum and blood eosinophil count (Busse et al., 2010; Laviolette et al., 2013).

Furthermore, similar results were reported in severe asthma patients. Pavord et al. (2012), Ortega et al. (2014), and Chupp et al. (2017) have reported that mepolizumab treatment was associated with lower rates of exacerbations and symptoms and with greater improvements in health-related quality of life compared with placebo among patients with severe eosinophilic asthma. Similarly, a meta-analysis by Farne et al. has revealed that anti-IL-5 reduced asthma exacerbations roughly by half (Farne et al., 2017). In addition, Cabon et al. have conducted an RCT and reported that mAbs targeting IL-5 significantly reduced blood and sputum eosinophil counts and attenuated bronchial submucosal eosinophils by approximately 50% in patients with eosinophilic asthma (Cabon et al., 2017).

However, no significant improvement in lung function, quality of life, and hospitalization rate was observed in the anti-IL-5 group. Anti-IL-5 therapy was associated with a mean difference of −0.01–0.03 L in pre-bronchodilator FEV_1_ compared with placebo. A change of 0.1 L from baseline in FEV_1_ has been described as a difference that patients can perceive (Donohue, 2005). The mean difference in SGRQ reduction between the anti-IL-5 and placebo groups was 0.29–2.05, while a threshold of 4 units is considered clinically significant (Jones, 2005). Likewise, other anti-inflammatory therapies for COPD, including macrolide antibiotics, have been reported to show similar results, i.e., significant reductions in exacerbation rate that were not associated with significant improvements in pre-bronchodilator FEV_1_ or health-related quality of life (Herath et al., 2018). A major therapeutic goal in COPD patients is to prevent or reduce future exacerbations (Dave and Arjun, 2021). Therefore, anti-IL-5 therapy can be considered for use in eosinophilic COPD patients due to the decrease in acute exacerbation rate. Based on the GOLD guidelines, cornerstone treatments such as LAMA, LABA, and ICS greatly improve lung function and the quality of life (Dave and Arjun, 2021). Additionally, the anti-IL-5 group was associated with a lower risk of severe adverse events than the placebo group. This result was consistent with that noted in previous phase 3 trials of benralizumab for severe, uncontrolled eosinophilic asthma (Bleecker et al., 2016; FitzGerald et al., 2016).

There was heterogeneity in the SGRQ total score. We speculate that the main source of this heterogeneity was the subjectivity of the scorer’s perception of the scale. In addition, a single scoring scale does not accurately reflect the true status of the quality of life. Heterogeneity also existed in the change from baseline of pre-bronchodilator FEV1. One possible reason might be that the measurement device or the professional level of the implementer may be different. Another reason may be that the education and cooperation level of COPD patients could influence lung function test results.

There are several limitations to this meta-analysis. First, among the RCTs admitted included in this meta-analysis, benralizumab failed to reduce the annual rate of acute exacerbation, whereas mepolizumab showed opposite results. The differences observed between benralizumab and mepolizumab might be due to the differences in sample sizes of the studies. In addition, owing to the limited original research, we could not perform subgroup analysis and the reliability of the conclusions inevitably decreased. Therefore, additional large RCTs assessing the efficacy of anti-IL-5 therapy (including benralizumab, mepolizumab, and reslizumab) in eosinophilic COPD patients are urgently needed. Second, although we conducted the comparison between anti-IL-5 therapy in eosinophilic COPD and in asthma, further RCTs that compare the anti-IL-5 therapy with ICS in eosinophilic COPD are needed, which may allow us to better determine the efficacy of anti-IL-5 therapy in eosinophilic COPD. Finally, all RCTs included in this meta-analysis were sponsored by a biopharmaceutical company.

## Conclusion

In this meta-analysis, we found that anti-IL-5 therapy significantly reduced the annual acute exacerbation rate and severe adverse events among eosinophilic COPD patients. In contrast, anti-IL-5 therapy did not improve lung function, quality of life, or hospitalization rate.

## Data Availability

The original contributions presented in the study are included in the article/Supplementary Material; further inquiries can be directed to the corresponding author.
