# Supplementary material for: Monoclonal Antibodies Targeting IL-5 or IL-5Rα in Eosinophilic Chronic Obstructive Pulmonary Disease: A Systematic Review and Meta-Analysis
Source: Front Pharmacol. 2021 Nov 2;12:754268. doi: 10.3389/fphar.2021.754268 (PMC8594629; doi:10.3389/fphar.2021.754268)
Supplement: Supplementary file 1 [file DataSheet1.DOCX]

**Supplementary**

**1. Supplementary Figures**


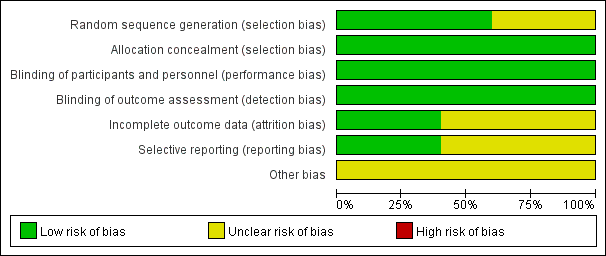


Supplementary Figure 1. Risk of bias graph presenting each risk of bias item as percentages across all included studies.


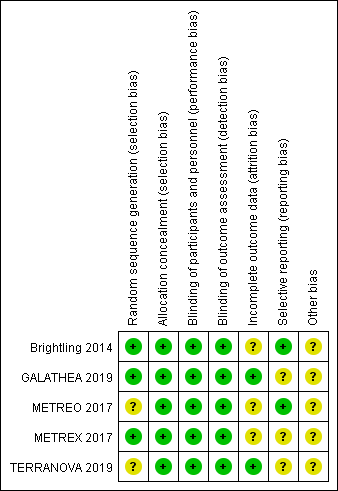


Supplementary Figure 2. Risk of bias summary for included studies, showing each risk of bias item for every included study.
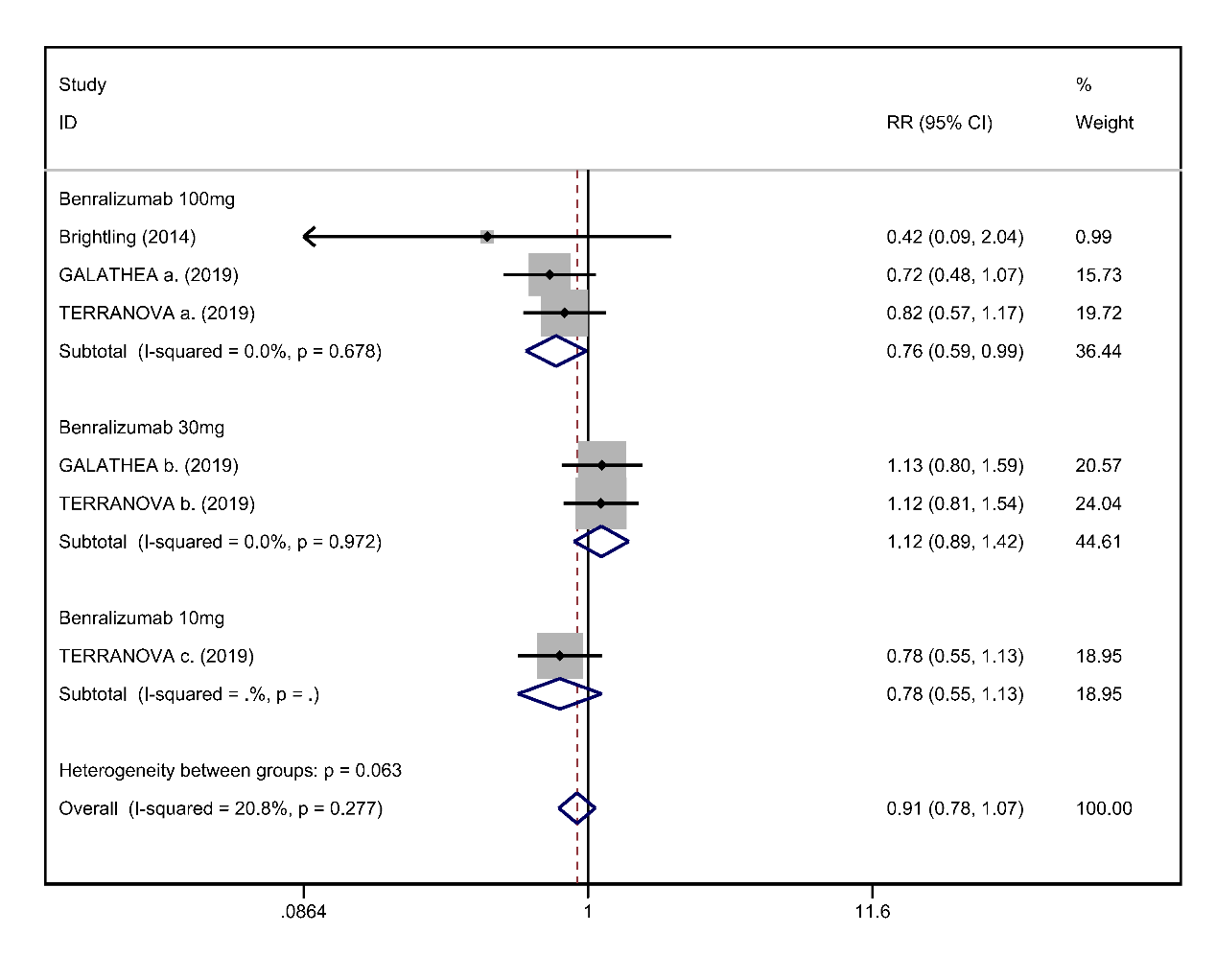
 Supplementary Figure 3. Forest plot of hospital admission rate for acute exacerbation in eosinophilic COPD patients with anti-IL-5 therapy versus placebo.
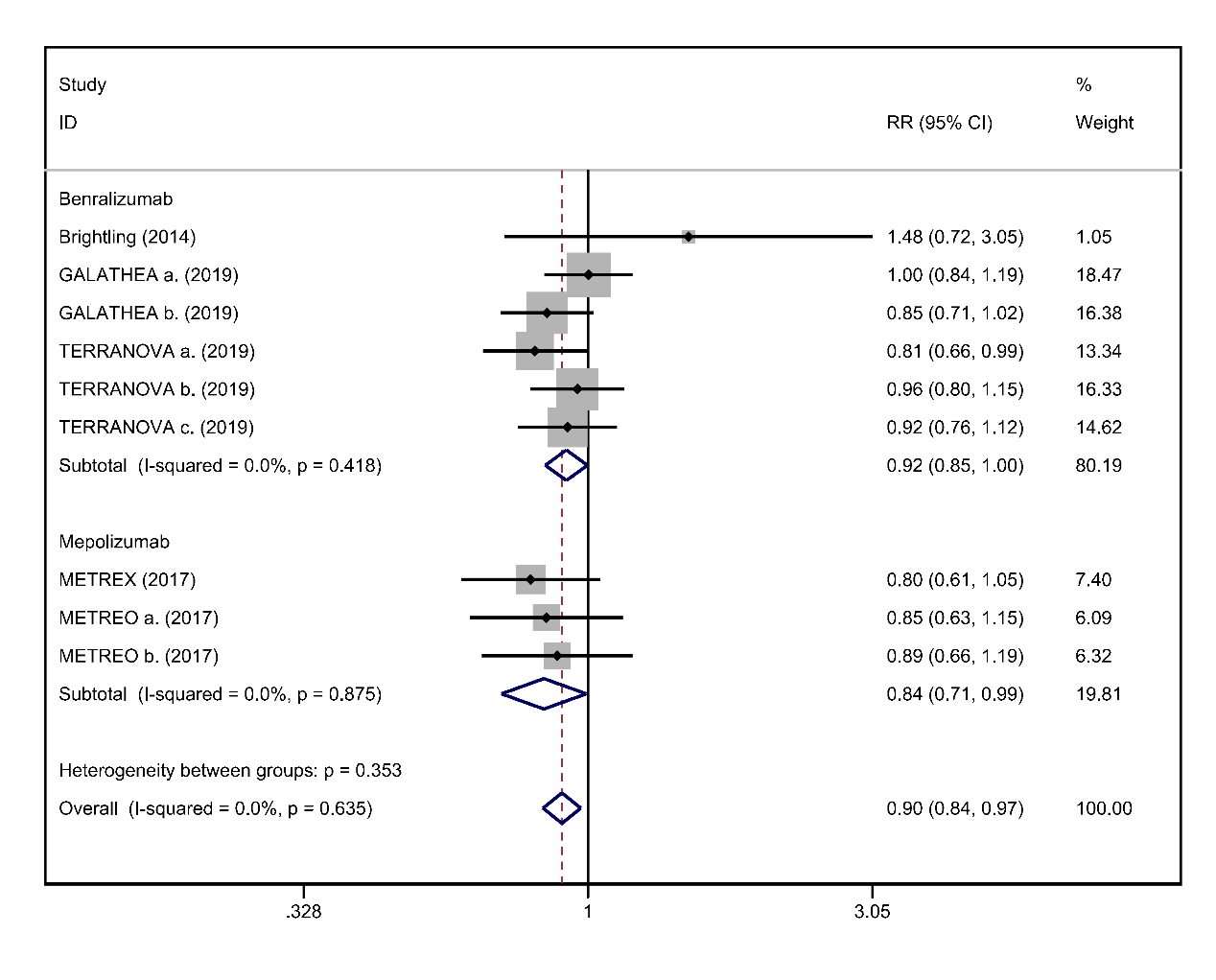


Supplementary Figure 4. Forest plot of severe adverse event in eosinophilic COPD patients with anti-IL-5 therapy versus placebo.

**2 Detailed search strategies**

**2.1 PubMed search strategy**

((("benralizumab"[Title/Abstract] OR "mepolizumab"[Title/Abstract]) OR "reslizumab"[Title/Abstract]) OR "antibodies, monoclonal"[MeSH Terms]) AND (((((((((("COPD"[Title/Abstract] OR "chronic obstructive pulmonary disease"[Title/Abstract]) OR "COAD"[Title/Abstract]) OR "chronic obstructive airway disease"[Title/Abstract]) OR "chronic obstructive lung disease"[Title/Abstract]) OR "airflow obstruction chronic"[Title/Abstract]) OR "airflow obstructions chronic"[Title/Abstract]) OR "chronic airflow obstructions"[Title/Abstract]) OR "chronic airflow obstruction"[Title/Abstract]) OR "COBD"[Title/Abstract]) OR "pulmonary disease, chronic obstructive"[MeSH Terms])

**2.2 Web of Science search strategy**

**主题:** (Chronic Airflow Obstruction) *OR* **主题:** (COBD) *OR* **主题:** (COPD) *OR* **主题:** (Chronic Obstructive Pulmonary Disease) *OR***主题:** (COAD) *OR* **主题:** (Chronic Obstructive Airway Disease) *OR* **主题:**(Chronic Obstructive Lung Disease)*OR* **主题:** (Airflow Obstruction, Chronic) *OR* **主题:** (Airflow Obstructions, Chronic) *OR* **主题:**(Chronic Airflow Obstructions) AND **主题:** (monoclonal antibodies) *OR* **主题:** (reslizumab) *OR***主题:** (mepolizumab) *OR* **主题:**(benralizumab)

**2.3 Cochrane Library search strategy**

ID Search

#1 MeSH descriptor: [Pulmonary Disease, Chronic Obstructive] explode all trees

#2 COPD

#3 Chronic Obstructive Pulmonary Disease

#4 COBD

#5 Chronic Airflow Obstruction

#6 Chronic Airflow Obstructions

#7 Airflow Obstructions, Chronic

#8 Airflow Obstruction, Chronic

#9 Chronic Obstructive Lung Disease

#10 Chronic Obstructive Airway Disease

#11 #1 OR #2 OR #3 OR #4 OR #5 OR #6 OR #7 OR #8 OR #9 OR #10

#12 MeSH descriptor: [Antibodies, Monoclonal] explode all trees

#13 benralizumab

#14 mepolizumab

#15 reslizumab

#16 #12 OR #13 OR #14 OR #15

#17 #11 AND #16

**2.4 Embase search strategy**

('chronic obstructive lung disease'/exp OR 'chronic airflow obstruction':ti,ab OR 'chronic airway obstruction':ti,ab OR 'chronic obstructive bronchitis':ti,ab OR 'chronic obstructive bronchopulmonary disease':ti,ab OR 'chronic obstructive lung disease':ti,ab OR 'chronic obstructive lung disorder':ti,ab OR 'chronic obstructive pulmonary disease':ti,ab OR 'chronic obstructive pulmonary disorder':ti,ab OR 'chronic obstructive respiratory disease':ti,ab OR 'copd':ti,ab OR 'lung chronic obstructive disease':ti,ab OR 'lung disease, chronic obstructive':ti,ab OR 'lung diseases, obstructive':ti,ab OR 'obstructive lung disease':ti,ab OR 'obstructive lung disease, chronic':ti,ab OR 'obstructive pulmonary disease':ti,ab OR 'obstructive respiratory disease':ti,ab OR 'obstructive respiratory tract disease':ti,ab OR 'pulmonary disease, chronic obstructive':ti,ab OR 'pulmonary disorder, chronic obstructive':ti,ab) AND ('monoclonal antibody'/exp OR 'antibodies, monoclonal':ti,ab OR 'antibodies, monoclonal, humanized':ti,ab OR 'antibodies, monoclonal, murine derived':ti,ab OR 'antibodies, monoclonal, murine-derived':ti,ab OR 'antibody, monoclonal':ti,ab OR 'clonal antibody':ti,ab OR 'hybridoma antibody':ti,ab OR 'monoclonal antibodies':ti,ab OR 'monoclonal antibody':ti,ab OR 'benralizumab'/exp OR 'benralizumab':ti,ab OR 'fasenra':ti,ab OR 'medi 563':ti,ab OR 'medi563':ti,ab OR 'mepolizumab'/exp OR 'bosatria':ti,ab OR 'mepolizumab':ti,ab OR 'nucala':ti,ab OR 'sb 240563':ti,ab OR 'sb-240563':ti,ab OR 'sb240563':ti,ab OR 'reslizumab'/exp OR 'cinqaero':ti,ab OR 'cinqair':ti,ab OR 'reslizumab':ti,ab OR 'sch 55700':ti,ab OR 'sch55700':ti,ab) AND ('placebo'/exp OR 'placebo' OR 'placebo gel' OR 'placebos')
